# Feasibility of gene transfer with nonviral vectors in murine models of sepsis

**DOI:** 10.1186/cc12991

**Published:** 2013-11-05

**Authors:** Vanessa B Faiotto, Rodolfo ME Hubert, Devanira S Paixao, Gleice R Souza, Maiara ML Fiusa, Carolina Costa-Lima, Sara TO Saad, Joyce M Annichino-Bizzacchi, Erich V De Paula

**Affiliations:** 1 Faculty of Medical Sciences, University of Campinas, SP, Brazil

## Background

Although several target-specific therapies for sepsis failed to translate into clinical benefits during the last decades, the increasing knowledge about sepsis pathogenesis continues to reveal new therapeutic targets that could be explored in the future. One of the challenges of previous target-specific treatments for sepsis was the short half-life of agents, some in the range of minutes. Gene transfer strategies can overcome this limitation, by providing a platform for longer expression of secreted therapeutic proteins. On the other hand, the transient nature of sepsis precludes the use of gene transfer strategies leading to long-term expression such as viral vectors. In this context, the use of nonviral vectors emerges as an attractive strategy for the treatment of sepsis, provided that sufficient expression of any therapeutic gene can be obtained.

## Materials and methods

Two gene constructs were used to evaluate the feasibility of gene transfer in the endotoxemia model: a lacZ expression plasmid driven by the CMV promoter, and a coagulation factor IX expression plasmid with the hAAT liver-specific promoter. The latter was used as a reporter gene for secreted proteins. C57Bl/6 mice were challenged with LPS and gene transfer was performed 6 hours thereafter, so as to mimic the timepoint when sepsis treatments would be initiated. Fifty micrograms of plasmid were injected into the tail vein using hydrodynamic transfection. A less aggressive protocol, which could in principle be translatable to the clinical setting, was also tested. Gene expression was evaluated 72 hours after gene transfer by a blinded investigator.

## Results

Factor IX activity levels (FIX:C) were significantly lower in nontransfected LPS-challenged mice (*n *= 12) compared with nontransfected controls (*n *= 14), suggesting that endotoxemia decreases baseline FIX:C levels. Higher FIX:C levels (twofold higher than controls) were observed in control mice submitted to hydrodynamic transfection (*n *= 5), as expected. When gene transfer was evaluated in the context of sepsis, LPS-challenged mice (*n *= 9) presented 1.7-fold higher FIX:C levels than control mice (*n *= 12) (*P *< 0.01). Moreover, mice that were exposed to a less aggressive intravenous transfection protocol (*n *= 8) presented FIX:C levels that were 1.4-fold higher than controls (*P *= 0.04). Liver-expression of β-galactosidase also demonstrated the feasibility of gene transfer in LPS-challenged mice.

## Conclusions

Our results suggest that the cellular and molecular events of sepsis reproduced in the endotoxemia model could facilitate gene transfer, thus offering a unique opportunity for gene therapy with nonviral vectors, without the need for traumatic gene transfer protocols that would be required in other pathological conditions.

